# Use of routine health information systems data in developing and monitoring district and facility health plans: a scoping review

**DOI:** 10.1186/s12913-023-09914-6

**Published:** 2023-10-02

**Authors:** Elaine Byrne, Arthur Heywood

**Affiliations:** 1 HISP Centre, University of Oslo, Oslo, Norway; 2 HISP Africa, Tanga, Tanzania

**Keywords:** Scoping review, Routine health information systems, RHIS, Planning, Monitoring, Supervision

## Abstract

**Background:**

Routine Health Information Systems data should be used in a systematic and institutionalised manner to support the making of plans, the monitoring of plans and in supportive supervision. To explore to what extent there is discussion about the linkage between planning, monitoring and supervision of sub-national programs using routine data we conducted a scoping review. The review question was: *How are routine health information systems used in developing and monitoring health plans at district and facility level?*

**Methods:**

From a search of Ovid Medline (all), EMBASE and Web of Science along with a review of grey literature and involving a number of key stakeholders in identifying any missing resources a total of over 2200 documents were reviewed and data from 13 documents were extracted.

**Results:**

Overall, there are many descriptions of how to implement and strengthen systems, ways to assess and improve data availability and quality, tools to improve the data use context, training in data use and mechanisms to involve stakeholders and strengthen infrastructure. However, there are gaps in examples of routine health data being used in the development, monitoring and supervision of plans at district and facility level.

**Conclusions:**

There appears to be no institutionalised obligation of planners to monitor plans, very little guidance on how to practically monitor programs and minimal discussion about how to use the routinely available data to supportively supervise the implementation of the plans. To overcome these shortcomings, we recommend that practical procedures to ensure linkage of existing district plans to regular monitoring of priority programs are institutionalised, that mechanisms for making managers institutionally accountable for monitoring and supervising these plans are put in place, and that practical guidelines for linking plans with routine health information system data and regular monitoring and supportive supervision are developed.

**Supplementary Information:**

The online version contains supplementary material available at 10.1186/s12913-023-09914-6.

## Background

In planning, managing, governing, decision making and delivering health services, programs and interventions, data informs decisions at all levels of the health system [1–3]. Routine Health Information System (RHIS) data should be used in a systematic and institutionalised manner to support the making of plans, the monitoring of plans and in supportive supervision. There are many tools and assessments to monitor and evaluate health information systems (HIS) strengthening interventions which rely on assessing data quality and data use. Many definitions and methods for assessing data quality exist (i.e., accuracy, reliability, precision, completeness, timeliness, integrity, and confidentiality) [4]. However, there is less consensus and fewer monitoring tools available for routine data use and very little discussion about the linkage between planning, monitoring and supervision of subnational programs using routine data.

In a recent scoping review [5] there were many examples of the use of DHIS2 (a RHIS platform used in over 70 Lower and Middle Income Countries (LMICs)) data in terms of program review and planning. The most common areas were in terms of developing periodic plans, for the monitoring and comparison of performance, for review meetings, and for use in reports. However in terms of planning, very little detail was given on how the DHIS2 informed the plans - in most documents there were simple statements made about DHIS2 data informing plans and in one case excerpts from the plan were presented [6]. However, no further detail was given on how action plans were previously used, not used or planned to be implemented.

It is not surprising then that a review of data use work practices, in Chinsali, Zambia (unpublished report by author AH), found that data from DHIS2 (the routine health information system in Zambia) is not being used to develop, monitor or evaluate the district and sub-district health performance plans. The annual plans in Zambia include the Reaching Every District (RED) plans. The RED approach [7] has five operational strategies developed through a facility/ district micro planning tool using routine data and has been implemented since 2003 in 53 LMICs [8] and adapted to country realities. However, in Zambia though the RED plans were found to be comprehensive and reflect priorities of the health sector, data is collected in a variety of formats and once developed, were not used. No monitoring took place and supervision did not use data. Additionally, in Chinsali, the key indicators in the RED plans were not matched to routine data collected at the facility level. This is not an exception; multiple studies show that very few districts or facilities have updated micro plans [9, 10].

Boerma et al. [11] note that despite a number of international and national monitoring and evaluation frameworks and guidelines, routine health data is not being used to monitor or evaluate performance plans. They also note that many LMICs face challenges in producing data that would be of sufficient quality to permit the regular tracking of progress in health interventions/services and strengthening health systems [11]. However, we have found no systematic review of the literature on whether and how routine health data are used in the development, monitoring and supervision of facility or subnational health plans. This scoping review will address this gap.

## Defining data use

As Byrne & Sæbø [5] note, data use is not easy to define, as both ‘data’ and ‘use’ can be conceptualised in many different ways. Jones [12] suggests distinguishing between “data in principle” (as they are recorded) and the “data in practice” (as they are used). There are also different conceptualisations of ‘use’ and consequently many different definitions of data use. In the DHIS2 information cycle (https://docs.dhis2.org/en/use/what-is-dhis2.html) data use is understood as the central component of a cyclical process that starts with decisions, goes through data collection, visualisation, dissemination, discussion and interpretation and then back to decisions and actions. Similarly, Nutley interprets data use in decision-making “ … as the analysis, synthesis, interpretation, and review of data for data-informed decision-making processes, regardless of the source of data” and therefore use “… goes beyond data reporting and passive dissemination of reports.” (13, p2). Nutley [13] goes on to categorise data use in terms of data and information regularly demanded, analysed, synthesised, reviewed and used in: (i) program review and planning, (ii) advocacy and policy development, and (iii) decision-making processes. Nutley doesn’t define each of these categories but classifies all three as the long-term outcomes of the use of data. We are particularly concerned in this review with data being used in (i) program review and planning: informing the development of plans, in monitoring and supervision, and in evaluations or review of the plans.

Given the divergence in defining data use, it is not surprising that definitions and methods for monitoring and measuring data use have posed challenges. Nutley and Li [4] note that data sharing, visualisation, dissemination, and review are often considered cases of data use. As a result, there are many different dimensions of data use that get measured, for example transparency, timeliness, visibility, accessibility, dissemination of information, calculation of key indicators, preparation of information products, and presentation of the achievement of targets [4]. In this review we focus on the use of routine data and information specifically for the making of plans and their monitoring and supervision at sub-national level in the health system. This emphasises continuity of data use in quantifying the key performance indicators in the initial plan, collecting quality data at a local level and using the same indicators for periodic monitoring by program managers and their supervisors doing performance assessment. This does not guarantee action, but ensures that high quality data is available, visible and shared throughout the planning and implementation cycle.

## Methods & analysis

The Joanna Briggs Institute Guidelines approach of Peters et al. [14] was followed in this review and included the following steps: defining and aligning the objective/s and question/s; developing and aligning the inclusion criteria with the objective/s and question/s; describing the planned approach to evidence searching, selecting, charting and summarising the evidence. The review question for this literature review was:

*How are routine health information systems used in developing and monitoring health plans at district and facility level?*

Databases searched included: Ovid Medline (all), EMBASE and Web of Science along with a review of grey literature such as documents from WHO, MEASURE, UNICEF, DHIS2 resources, as well as involving a number of key stakeholders in identifying any missing resources.

Inclusion criteria were that the documents describe how RHISs are used or should be used in developing and monitoring their annual health plans. Exclusion criteria were:

1. Articles that mention challenges/ concerns with the development or monitoring of health plans only.
2. Articles that do not use routine data to develop or monitor health plans, but conduct a particular survey or research to monitor plans.
3. Documents that are theoretical/conceptual only with no examples of use in practice.
4. Non-English language studies.

All types of studies were included, as well as original research and reviews. No quality review was conducted as within a scoping review the complete landscape of publications is to be included regardless of quality. However, the article needed to describe the methodology and findings in sufficient detail to be informative, such as in terms of process, content, or findings.

Search terms included: ‘routine health information system(s)’ AND ‘plan’ OR ‘micro-plans’ AND ‘monitoring’ (see annex 1 for search strings). As the RED strategy is particularly relevant to the review question the same databases were also searched independently for any article that included ‘Reaching Every District’, ‘RED’ or ‘Reaching Every Child’.

All results were imported into Covidence systematic review software^1^ and duplicates removed. This search was updated periodically after the project start date and included the articles or documents retrieved through snowballing or from stakeholders. Included documents were validated by consulting with expert stakeholders to check for any missing relevant documentation. Identified sources of evidence underwent a two-level review process: a title and abstract review, and a full-text review. Data was charted against a number of criteria: Title of article/publication, Lead author, Year, Journal/publication outlet and Country of study. Further details included: practical examples of M&E of action plan, how M&E was implemented, at what level M&E took place, whether RHIS was highlighted as main source of data, whether a framework/model of M&E was presented, description of framework/mode, how M&E is related to a plan, what plan and what the main purpose of M&E.

**Fig. 1:**
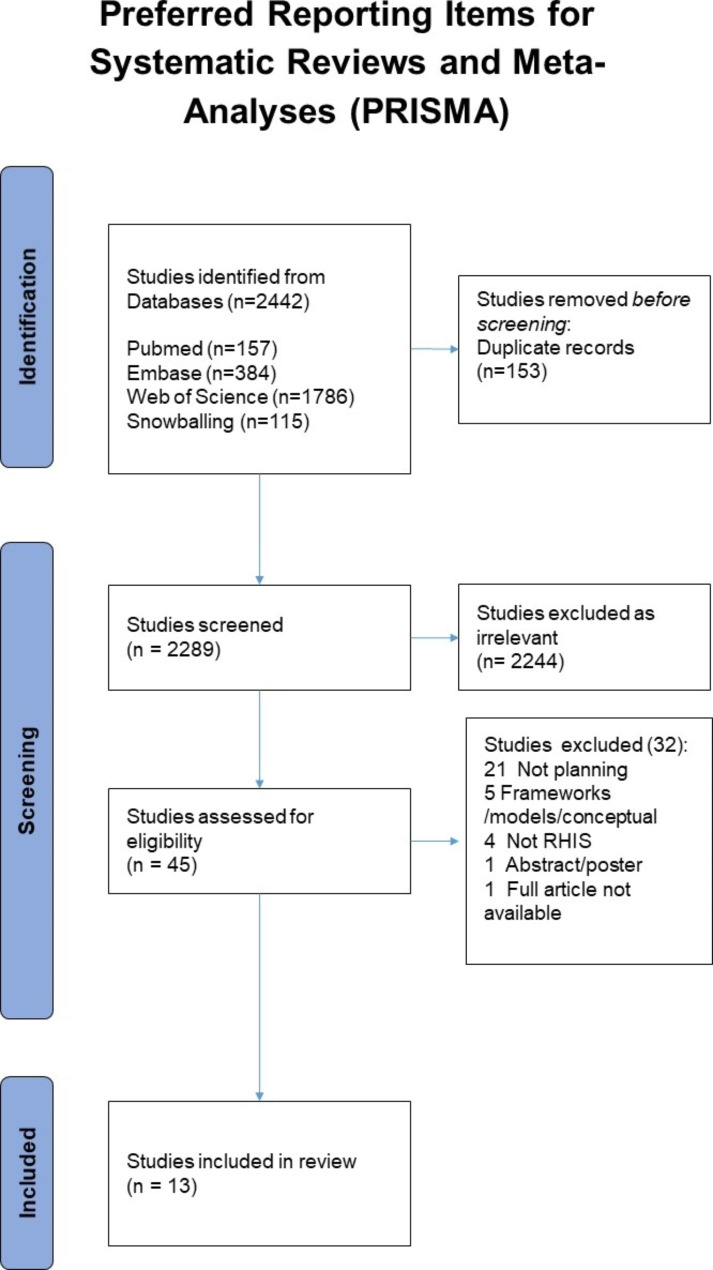
PRISMA indicating 13 articles included

A total of 2442 articles were imported, 153 duplicates removed and 2289 articles’ title and abstract reviewed. Forty-five full texts were reviewed and 32 excluded with the main reasons being the lack of focus of planning. Data from thirteen articles was extracted (Fig. 1). Both authors screened, reviewed and extracted at all stages. Both authors have decades of experience in RHIS in developing countries at district level and below, as well as in researching and writing about data and information use. Conflicts were resolved between the two authors (44 articles were discussed at title and abstract stage and 11 at full text review); most of the conflicts were over level of detail required for the article for it to be included rather than relevance. The department research group was available to arbitrate if it had been needed, but this was not required. The list of articles included and preliminary findings were shared with stakeholders from global health institutions such as UNICEF, WHO and RHINO, the health information systems research group at University of Oslo, and the Global Health Information Systems Programme network.

## Results: description of documents

A total of 13 documents were included at full extraction stage (Table 1). Most of the documents [10] were published between 2013 and 2019, with 3 each published in 2013 and 2016. The 13 documents were published in 8 different journals; journals that published more than one of the 13 included studies were Health Policy and Planning (4 articles), BMC Health Services Research (2 articles), and Health Research Policy and Systems (2 articles). Seven of the articles included studies conducted in Africa and 3 in Asia. The remaining 3 studies were done in South America, in the Middle East and in LMICs in general.

**Table 1:** Articles included at full text extraction stage

Lead author	Title	Publication outlet
Abdi, Z. et al. (2019)	Developing a framework for the monitoring and evaluation of the Health Transformation Plan in the Islamic Republic of Iran: lessons learned	East Mediterr Health J
Bernardi, R. (2017)	Health Information Systems and Accountability in Kenya: A Structuration Theory Perspective	Journal of the Association for Information Systems
Chan Soeung, S. et al. (2013)	From reaching every district to reaching every community: analysis and response to the challenge of equity in immunization in Cambodia	Health Policy and Planning
Chaulagai, C.N., et al. (2005)	Design and implementation of a health management information system in Malawi: issues, innovations and results	Health Policy and Planning
Cibulskis, R.E. et al. (2002)	Information systems for health sector monitoring in Papua New Guinea	Policy and Practice
Enkhtuya, B. et al. (2009)	Reaching every district - development and testing of a health micro-planning strategy for reaching difficult to reach populations in Mongolia	Rural and Remote Health
Mutale, W. et al. (2013)	Improving health information systems for decision making across five sub-Saharan African countries: Implementation strategies from the African Health Initiative	BMC Health Services Research
Nabyonga-Orem, J. et al. (2016)	Harmonisation and standardisation of health sector and programme reviews and evaluations - how can they better inform health policy dialogue?	Health Research Policy and Systems
Nicol, E. et al. (2017)	Perceptions about data-informed decisions: an assessment of information-use in high HIV-prevalence settings in South Africa	BMC Health Services Research
Nutley, T. et al. (2013)	Impact of a decision-support tool on decision making at the district level in Kenya	Health Research Policy and Systems
Sellera, P.E.G. et al. (2019)	The Implementation of the Monitoring and Evaluation System of the State Health Secretariat of the Brazilian Federal District (SHS/DF)	*Ciência & Saúde Coletiva* (Science and Public Health)
Wagenaar, B.H. et al. (2016)	Using routine health information systems for well-designed health evaluations in low- and middle-income countries	Health Policy and Planning
Wickremasinghe, D., et al. (2016)	District decision-making for health in low-income settings: a systematic literature review	Health Policy and Planning

## Results: content of documents

The documents are described in two subsections based on the two overarching categories that emerged from the included articles: (i) results from country evaluations/assessments of M&E systems and processes, and; (ii)evaluations/reviews of plans. In terms of the exclusion criteria we excluded theoretical/conceptual articles/reports that had no practical base. Examples of those excluded are:

- a logic model for improving the use of health data for health system strengthening (HSS) with recommendations that affect the use of data in decision making [15].
- a correspondence article on planning, implementation, monitoring, and evaluation of integrated health services which emphasises the importance of strong monitoring and evaluation (M&E) systems [16].
- a country-led platform for information and accountability that provides guidance to countries and partners for strengthening monitoring, evaluation and review of national health plans and strategies, but no specific plan is being monitored [17].
- use of RHIS to evaluate HSS interventions, with a good description of indicator selection, but not linking use of RHIS to evaluate plans [18].
- an Organizing Framework for a Functional National HIV Monitoring and Evaluation System but without detailed guidance on how to operationalize the system [19].

However, in the discussion section of this article we return to some of these conceptual models for their potential to inform guidelines on local level planning, monitoring and supervision of action plans.

### Evaluations/Assessments of M&E systems and processes

In a study in South Africa on perceptions about data-informed decisions for HIV, Nicol et al. [20] explore the challenges in relation to data use and note that organisational and capacity issues need to be addressed before information will be used. This includes the development of a culture of information use, trust in the data, and capacity to analyse, interpret and use information. They suggest that “Facility and program managers should be provided with opportunities for capacity development as well as practice-based, in-service training, and be supported to use information for planning, management and decision-making” [20, p.765]. However, there are no practical guidelines or suggestions on how this is to be achieved. They observe that there are mechanisms and processes in place to promote use of information (performance meetings, access to routine reports, directives/ standard operating procedures (SOPs), monthly targets, existence of information), but these are not being used (or are ‘selectively used’).

Nabyonga-Orem, J. et al. [21] look at the harmonisation and standardisation of the health sector and programme reviews in the WHO African Region. They highlight the main challenges in terms of performance assessment (weak institutional capacity for M&E, desynchronised planning timeframes, inadequate time allocated for comprehensive performance assessments, weak follow-up mechanisms, lack of stakeholder engagement and divergent political agendas). They call for the standardisation and institutionalisation of performance assessments, but give no detail on how this can be done and do not link it with planning and a comprehensive M&E framework.

In Malawi, Chaulagai et al [22] reviewed the District Implementation Plan (DIP) process and described the stages of getting baseline data to set priorities and targets in the DIP. They conclude that most DIPs are vague and that it is hard to track implementation status and results. There were some attempts to computerise the DIP process so that there are links with the routine data collected for monitoring the plan, but this resulted in only the person entering the data being involved in the planning and monitoring process. The purpose of the DIP process was to enable the allocation of resources based on performance, and so that facilities and districts could compare or rank their performance in relation to other facilities of districts. The DIPs should also be accompanied by a decentralised system of quarterly feedback, supportive supervision visits and annual reviews. However, there is no detailed description of a M&E plan or process on how the DIP should be monitored or supervised. However, some good practices were identified: gaining consensus on indicators and tools, skills training on utilising existing data to calculate indicators and management of health services, establishing regular meetings and reporting (quarterly management and annual performance reviews at all levels) and the development of routine monitoring and guidelines for an annual health sector joint review. They conclude that there was overall little improvement in the use of data in rationalising decisions and that “no matter how good the design of an information system, it will not be effective unless there is internal desire, dedication and commitment of leadership to have an effective and efficient health service management system” [22, p.375].

In other articles there is mention of data being used in planning and monitoring, but the articles are not particularly informative in terms of practical examples or lessons learned. For example, a theoretically informed review of the Kenyan HIS and accountability, includes some mention of use of data in monitoring plans, but it is rather vague [30]; a M&E system at national level in Brazil highlights the importance of integrating all the M&E tools and plans across departments and units, but does not focus on how this was done or how the integrated plans were monitored [23], and; the development of a framework for the M&E of a National plan in Iran indicates that numerous surveys as well as RHIS would be needed to monitor the plan and even then there would be gaps in data needed to monitor the plan [24]. Similarly, a review of 5 HSS changes made note that a common evaluation framework of HIS strengthening was used, but it is not described [25]. The authors note that there is hope that this will assist in “… linking HIS with decision making, and its impact on measures of health system outputs and impact” [25, p.1], but no examples are given. Exploring other changes made to HIS for improving use in decision-making, Nutley et al. [26] look at the impact of the District Health Plan decision-support tool on decision making at the district level in Kenya, but there is little detail given on the tool and there is no link to a plan or monitoring of the plan. In fact, the 11 review questions of the HIV programme included in the tool works in parallel rather than as part of an integrated M&E system.

Some of the other included articles reviewed indicate that RHIS data could be used in monitoring programmes even if it of variable quality [27]. Cibulskis et al. [28] argue that even imperfect routine health data can be used whilst simultaneously working on the quality and completeness. This will encourage ‘a more methodical approach to planning and monitoring services’ [28, p.752] and lead to improved quality of data that can be used on a routine basis to monitor performance.

Overall the published evaluations/assessments of M&E systems and processes do not reveal a practice of using RHIS data in the planning, monitoring and supervision process. Some of the key findings could inform the implementation of such a system, namely, the need to develop organisational and institutional capacity [20], the standardisation and institutionalising of routine performance assessment [21] and the commitment of leadership to effective and efficient service management [22].

### Evaluations and reviews of plans

One example of monitoring facility level plans was in the study by Enkhtuya et al. [8] when reviewing the national RED planning in one district. The district and family practitioners were able to map areas of low coverage, undertake barrier analysis, and provide detailed costed activities required to reach these populations. The RED M&E plan includes supportive supervision (such as local problem solving, involvement of community), micro-planning activities and budget requests through normal annual operational planning and budgeting processes. Overall though the reviewers concluded that there is a need to change the RED approach as local area data and knowledge are currently absent in both plans and supportive supervision. Changing the style and approach to planning requires a change in management - moving from supervisor as inspector to supporter. In fact Chan Soeung et al. [29] draw a similar conclusion on community level involvement in terms of planning, recognising that “a shift in planning focus is needed from a district-wide perspective down to a facility- and community-level system of analysis and operations” [29, p.532]. However, the Chan Soeung et al. [29] study was on equity and not planning and so provides little insight into using RHIS for planning, monitoring and supervision.

The only other article that looked specifically at decision-making and planning was a literature review of decision making for health in LMICs by Wickremasinghe et al. [30]. They found twelve examples of tools to assist district-level decision-making. The major use of data for decision-making was to identify priorities, and in developing an action plan to address those priorities. Four of the studies included steps for reviewing or monitoring the plans with HIS data, but “… there was limited evidence about their sustained impact on district level decision-making and whether they have led to changes in resource allocation patterns” (30, pii23). In terms of lessons around data use that could be used in planning, monitoring and supervision, there were three features that kept recurring: relevance depended on data quality, for consensus a structured decision-making process is needed and, that communities need to be included in the decision-making process.

Overall these two articles indicate the need for local level planning that involves communities and the review article indicates the importance of data quality and a structured process for decision making.

To consolidate the contributions of the included articles in this review we map the content of the articles extracted against the 8 domains of the MEASURE Evaluation Logic Model [4]

i Assessing and improving the data use context
ii Engaging data users and data producers
iii Improving data quality
iv Improving data availability
v Identifying information needs
vi Building capacity in data use core competencies
vii Strengthening the organisation’s infrastructure
viii Monitoring, evaluating, & communicating successes. (Table 2)

**Table 2:** Article content mapped to MEASURE Evaluation logic model domains

MEASURE Evaluation logic model domains	Content of included articles related to domain
Assessing and improving the data use context	• Assess and improve the data use context/data culture [15] • Organisational and capacity issues such as the development of a culture of information use [20] • Requires an institution taking responsibility and accountability [16, 31] • Stakeholders need to make the link between data collection and strategic plans, operational plans and program plans [16] • Stakeholders and decision-makers need to place value on data they use in decision making [15] • ‘The internal desire, dedication and commitment of leadership’ [22] • Consensus on a structured decision-making process [30] • Standardisation, institutionalisation and coordination of planning, M&E, assessments and follow up mechanisms [21] • “As institutionalised as stocking a pharmacy or immunising a child” [25] • Problem-solving planning methodology progressing from health mapping to barrier analysis, to activity planning and costing and finally to monitoring and evaluation [8]
Engaging data users and data producers	• Engage with other stakeholders: data users and data producers [15] • Communities need to be included in the decision-making process [30] • Shifting emphasis to the health centre and community [8, 29]
Improving data quality	• Improve data quality [15, 18] • Relevancy depends on quality [30] • Developing trust in data [20] • Improve existing systems whilst using even imperfect routine health data [28]
Improving data availability	• Improve data availability [15]
Identifying information needs	• Identify information needs [15, 17]
Building capacity in data use core competencies	• Build capacity in data use core competencies [15] • develop capacity to analyse, interpret and use information [20] • Re-orientation of management approaches from ‘inspection’ to supportive supervision [8]
Strengthening the organisation’s infrastructure	• Improve organisation’s data demand and use infrastructure [15] • Mechanisms to improve practice gaining consensus on indicators and tools, skills training on using data to calculate indicators and management of health services, establishing regular meetings and reporting, the development of routine monitoring and guidelines for an annual health sector review [22]
Monitoring, evaluating, & communicating successes	• Monitor, evaluate, and communicate results of data use interventions [15] • Development of communication strategies and health networks that enable providers to adjust service delivery according to the needs of vulnerable population [29]

## Discussion

This study looked at the link between planning and monitoring at subnational level using routine health information and found that there were surprisingly few articles that looked at the concept and even fewer that described the practicalities of implementing the links. While there are a number of high-level descriptions of what should be done to develop a culture of information use, most were very vague and there were very few details of how this process can be institutionalised, how staff are held accountable for implementing their plans or how to actually monitor the plans made. What we found missing was practical guidelines or examples on how to institutionalise regular, structured data use for monitoring program performance at service delivery level and on how to ensure informed discussion and interpretation by lower level stakeholders.

However, there are some tested existing frameworks, such as PRISM, health information system strengthening model (HISSM), MEASURE Evaluation Logic Model for improving data use [4], on the use of routine data in monitoring as well as global strategies that support the development and review of lower level/micro plans. We did not find any articles using these in the literature we reviewed, but the frameworks could be used in conjunction with the findings from our review to inform development of improved guidelines for planning, monitoring and supervision at local level.

Nutley and Li [4] reviewed the main assessments and tools used for data use. They include a review of data use in the HISSM and the MEASURE Evaluation Logic Model for improving data use. The MEASURE Evaluation Logic Model describes specific activities (8 domains) and interventions needed to improve the use of health data for improved health programs and policies. This model builds on the HISSM by providing specific and detailed ways to support the use of HIS data. There is no specific reference to use of data in developing, monitoring and supervising plans made in any of the models, but Nutley and Li [4] look at various assessment tools and map them according to the dimensions of data use (data quality, health statistics, information products, data review, advocacy, decision and action). None of these tools are specific to data use and action planning. Nutley and Li conclude that “Few tools that measure the outcome of data use for improved health program performance exist. …. Better measures of the outcome of data use are needed, along with ways to easily track the health program and health system outcomes associated with decisions that are implemented.“ [4, p.32]. Our review confirms this conclusion.

There are two domains of the MEASURE Evaluation Logic Model [4] (Table 2) that could be expanded based on the information we extracted from the articles, namely assessment and improving the data use context and strengthening the organisations infrastructure. The domain of assessing and improving the data use context is often referred to as developing a culture of information use and we are reminded by Reynolds et al. [16] and Bernardi [31], that such a culture requires an institution to take responsibility and accountability. More detail on how to achieve improvements in the context include: stakeholders linking data collection and strategic plans, operational plans and program plans [16]; stakeholders placing value on the data [15] and showing commitment [22], and; gaining consensus on a structured decision-making process [30]. All of this requires standardisation, institutionalisation and coordination of planning, M&E, assessments and follow up mechanisms [21]. As Mutale et al. [25] note, data use in planning, monitoring and supervision needs to be as institutionalised as stocking a pharmacy or immunising a child. In terms of engaging data users and data producers Wickremasinghe et al., [30] remind us of the importance of including the community in the decision-making process as key data users and producers. Shifting planning emphasis to the health centre and community is also raised [8, 29].

In relation to data quality, most articles highlighted how important data quality was if it was to be used. Many tools have been developed to improve data quality including minimal data entry and automated graphs [26]. Equally important is that the data is trusted by users [20], but this is often subjective and goes beyond data quality. Cibulskis et al. [28], and Wagenaar et al. [27], emphasise that we should not wait until data quality is ‘perfect’ before using the data, as using the data is necessary to improve the system. Wagenaar et al. [27] note that RHIS data are often superior to intermittent community sample surveys that can have data delays far worse than RHIS and are costly to conduct. Whilst acknowledging concerns regarding completeness, timeliness, representativeness and accuracy of routine data, they note that with increased use, regular data quality monitoring and management and information technology, RHIS data could become the new ‘gold standard’ for health programme evaluations. There are still existing challenges with RHIS such as reliable population data, challenges to including data on the excluded populations and on neglected services.

There are many examples given of mechanisms or activities to strengthen the organisational infrastructure. Chaulagai et al. [22] give a comprehensive list, and Nicol et al. [20] give examples of key issues they found to facilitate a supportive organisational structure. These include, but are not limited to:

- Involvement of stakeholders to gain consensus on indicators and tools,
- skills training on using existing data to calculate indicators and management of health services,
- establishing regular local meetings for discussion, interpretation and feedback of data,
- the development of guidelines for routine monitoring and periodic program reviews,
- standardising and institutionalising the planning and monitoring process through agreed strategies, SoPs and guidelines.

However, there is no mention of the need to institutionalise use of routine data to develop and monitor annual plans at sub-national level nor how the data and plans can be used for supportive supervision or to hold stakeholders accountable for the implementation of the plans.

In a commentary on the historical evolution of M&E Thomas et al. [32] note that M&E for health in LMICs has advanced from an emerging discipline to one that adheres to standards and is systematised and mainstreamed across programmes. They also note that significant capacity has been developed and resources allocated to collecting and using data, enabling a move away from paper-based to electronic systems. What is interesting are the tensions that exist with these changes:

- A single, unified country system versus accountability and control by a variety of parallel diseases/ programmes or particular donors;
- The desire for a shared integrated system versus the desire for specific outcomes.
- Country autonomy versus donor control.

If we are to see more examples of RHIS use in planning, monitoring and evaluation, these tensions need to be addressed by creating a supportive context for effective data use. What is interesting for the authors is that, despite the growth of this discussion in the field, there is a lack of debate in the literature on whether paper-based or electronic health information systems facilitated or inhibited data use. In a previous review on data use more generally, Byrne & Sæbø [5] noted that a number of articles illustrated paper-based data use practises operating parallel to the routine official DHIS2 data practises. We specifically did not include or exclude paper or electronic systems as we wanted to know if data, however collected or generated, was being used in the development and monitoring of local plans. We found that neither digital nor paper- based data was routinely used.

## Conclusion

Fundamentally what emerges from this review is that the main obstacle to using routine data in monitoring and supervision of annual plans is the weak institutionalisation of the planning and monitoring processes and lack of clarity on the organisational procedures needed for this to occur at sub-national level. Solving this challenge requires a decentralised data use vision by national leaders that links annual planning to regular monitoring with routine data. This should promote institutional commitment to local data use and empower data governance at all levels.

The basic premise of effective decentralised planning is that district level plans should be regularly monitored by the planners themselves using indicators from routine data, with their implementation supervised by immediate managers [33, 34] using the same indicators. Our review has found that this linkage does not exist in the published literature. There appears to be no institutional obligation of planners to monitor (micro) plans, very little guidance on how to practically monitor programs in an integrated manner and minimal discussion about how to use the routinely available data to supervise the implementation of the plans.

Overall, there are many descriptions of how to implement and strengthen systems, ways to assess and improve data availability and quality, tools to improve the data use context, training in data use and mechanisms to involve stakeholders and strengthen infrastructure. However, there are massive gaps in the literature of good data use cases or examples of where routine health data is used in the development, monitoring and supervision of plans at district and facility level.

Likewise, there are gaps in terms of written guidelines on how this should happen. The RED approach comes the closest to describing the expected links between planning, monitoring and supervision: it clearly links facility micro-planning, local monitoring of implementation and supervision [8, 29] and provides some instruction on how to implement the various steps. However, global RED guidance lacks practical details and it seems they have not been adapted by countries to ensure local implementation, particularly around data collection and use. Given the extent to which RED has been supported and implemented globally, it offers a well-known entry point to establish those links and provide practical guidelines on using RHIS to develop and monitor district and local action plans. A less verticalized and more integrated RED approach would be required. Consolidating the information on M&E from the reviewed documents and mapping to the MEASURE Evaluation logic model domains could be a useful starting point in enhancing practical guidelines on how to develop and monitor local action plans using routine data.

It is possible that some relevant documents may be published in journals not indexed on the databases searched or that the key experts consulted were not aware of guidelines available. We are therefore not concluding that there are no other examples or guidelines that exist, but that these examples or guidelines are not readily available. The main gaps that need to be addressed are:

- Practical procedures to ensure linkage of existing district plans to regular, institutionalised monitoring of priority programs using key performance indicators.
- Mechanisms for making managers institutionally accountable for regularly monitoring and supervising these annual plans using routine data.
- Developing practical guidelines for linking plans with RHIS data and linking plans with regular monitoring and supportive supervision.

### Electronic supplementary material

Below is the link to the electronic supplementary material.

Supplementary Material 1

Supplementary Material 2

## Data Availability

All data generated or analysed during this study are included in this published article.

## Footnotes

1 Covidence systematic review software, Veritas Health Innovation, Melbourne, Australia. Available at www.covidence.org.

## Abbreviations

DHIS2: District Health Information Software 2
DHP: District Health Plan
DIP: District Implementation Plan
HIS: Health Information System
HISSM: Health Information System Strengthening Model
HSS: Health System Strengthening
LMICs: Lower and Middle Income Countries
M&E: Monitoring and Evaluation
PRISM: Performance of Routine Information System Management
RED: Reaching Every District
RHIS: Routine Health Information Systems
SOPs: Standard Operating Procedures
UNICEF: United Nations Children’s Fund
WHO: World Health Organisation
