# Supplementary material for: Use of routine health information systems data in developing and monitoring district and facility health plans: a scoping review
Source: BMC Health Serv Res. 2023 Oct 2;23:1049. doi: 10.1186/s12913-023-09914-6 (PMC10544391; doi:10.1186/s12913-023-09914-6)
Supplement: Supplementary file 2 — Supplementary Material 2 [file 12913_2023_9914_MOESM2_ESM.docx]

| **Pubmed  - advanced search** |  |
| --- | --- |
| "Health Information Systems"[Mesh] OR “health information system*”[Text Word] OR HIS[tiab] OR hmis[tiab] OR rhis[Text Word] |  |
| plan [Text Word] OR micro-plan [Text Word] OR “micro plan” [Text Word] OR "Health Plan Implementation"[Mesh] |  |
| Monitor[Text Word] OR Monitoring[Text Word] |  |
| 1 AND 2 AND 3 | 157 |
| **EMBASE  - quick search** |  |
| ‘health information system*’ OR HIS OR hmis OR rhis |  |
| plan OR micro-plan OR ‘micro plan’ |  |
| Monitor OR Monitoring |  |
| 1 AND 2 AND 3 | 566 |
| Limit to SOURCES to Embase only excl. Medline | 384 |
| **WEB OF SCIENCE-  Select Editions: SCIENCE AND SOCIAL SCIENCE CITATION INDEXES only** |  |
| "Health Information Systems" OR “health information system*” OR HIS OR hmis OR rhis |  |
| plan  OR micro-plan  OR “micro plan” |  |
| Monitor OR Monitoring |  |
| 1 AND 2 AND 3 | 1830 |
| Limit to articles or review articles in English | 1786 |

**Annex 1: Search strings**
